# Beyond Step Count: Are We Ready to Use Digital Phenotyping to Make Actionable Individual Predictions in Psychiatry?

**DOI:** 10.2196/59826

**Published:** 2024-08-05

**Authors:** Abigail Ortiz, Benoit H Mulsant

**Affiliations:** 1 Department of Psychiatry Temerty Faculty of Medicine University of Toronto Toronto, ON Canada

**Keywords:** digital phenotype, digital phenotyping, prediction, predictions, mental health, mental illness, mental illnesses, mental disorder, mental disorders, US National Institute of Mental Health, NIMH, psychiatry, psychiatrist, psychiatrists

## Abstract

Some models for mental disorders or behaviors (eg, suicide) have been successfully developed, allowing predictions at the population level. However, current demographic and clinical variables are neither sensitive nor specific enough for making individual actionable clinical predictions. A major hope of the “Decade of the Brain” was that biological measures (biomarkers) would solve these issues and lead to precision psychiatry. However, as models are based on sociodemographic and clinical data, even when these biomarkers differ significantly between groups of patients and control participants, they are still neither sensitive nor specific enough to be applied to individual patients. Technological advances over the past decade offer a promising approach based on new measures that may be essential for understanding mental disorders and predicting their trajectories. Several new tools allow us to continuously monitor objective behavioral measures (eg, hours of sleep) and densely sample subjective measures (eg, mood). The promise of this approach, referred to as digital phenotyping, was recognized almost a decade ago, with its potential impact on psychiatry being compared to the impact of the microscope on biological sciences. However, despite the intuitive belief that collecting densely sampled data (big data) improves clinical outcomes, recent clinical trials have not shown that incorporating digital phenotyping improves clinical outcomes. This viewpoint provides a stepwise development and implementation approach, similar to the one that has been successful in the prediction and prevention of cardiovascular disease, to achieve clinically actionable predictions in psychiatry.

“It is difficult to make predictions, especially about the future” [1], as Yogi Berra stated.

Thirty years after the US National Institute of Mental Health (NIMH) declared the final decade of the 20th century to be “the Decade of the Brain,” Tom Insel, the NIMH director at that time acknowledged,

I spent 13 years at NIMH really pushing on the neuroscience and genetics of mental disorders, and when I look back on that I realize that while I think I succeeded at getting lots of really cool papers published by cool scientists at fairly large costs—I think $20 billion—I don’t think we moved the needle in reducing suicide, reducing hospitalizations, improving recovery for the tens of millions of people who have mental illness.

2

A challenge contributing to this issue is that day-to-day clinical interactions and decision-making processes in psychiatry remain fundamentally the same as they were 50 years ago. Decision-making is still based on binary (present/absent) diagnoses inferred from clinical symptoms assessed during an interview at a single time point. Once a diagnosis has been made, treatment focuses on managing acute symptoms, with simple long-term strategies (eg, “what gets you well, keeps you well”) and reactive handling of adverse outcomes (eg, readmission following a suicide attempt). Moreover, despite extraordinary empirical advances in psychopharmacology [3-5], all psychotropic medications help a subset of patients. Thus, most psychiatrists still follow a trial-and-error approach, which can take an inordinate amount of time [6]. Once patients are stable, the traditional model of clinical monitoring typically involves monthly visits that are either too infrequent or too frequent given the labile nature of mental disorders.

Some models for mental disorders or behaviors (eg, suicide) have been successfully developed and they allow predictions at the population level [7,8]. However, current demographic and clinical variables are neither sensitive nor specific enough for making individual actionable clinical predictions. Using suicide as an example, a recent meta-analysis concluded that predictive ability has not improved across 50 years of research [9]. This is in part because these predictive models are still solely based on sociodemographic and static descriptive clinical variables. For instance, an older, White man, with depression and alcohol use may have a 100-fold higher likelihood of killing himself during the next year than somebody in the general population. Unfortunately, this astounding relative risk (100) means that the likelihood that this single patient kills himself during the next year is 10/1000 (1%) rather than 10/100,000. Psychiatrists cannot hospitalize 100 patients for one year to save one life. Using larger datasets with more demographic and clinical variables may improve the precision of these population-based models, but they are unlikely to impact individual clinical outcomes.

A major hope of the “Decade of the Brain” was that biological measures (biomarkers) would solve these issues [10,11] and lead to precision psychiatry [12]. A variety of biomarkers have now been reliably associated with mental disorders and their outcomes [13]. However, as models based on sociodemographic and clinical data, even when these biomarkers differ significantly between groups of patients and control participants, they are neither sensitive nor specific enough to be applied to individual patients [14-16]. Typically, a quarter to a third of the patients have normal values and a quarter to a third of the controls have pathologic values [17]. New models that will integrate sociodemographic, clinical, and biological data are being developed, but they have not yet been shown to improve clinical outcomes [15,16,18,19]. The field of preventive psychiatry still lacks an understanding of the complex mechanisms underlying mental disorders and their treatment, in contrast to the well-established pathophysiologic models in cardiology or oncology, which link risk factors to outcomes and have led to reductions in mortality related to heart disease, stroke, or cancer [20,21].

Looking back, we believe that our inability over two or three “decades of the brain” to bridge the gap between biology and clinical symptoms is due to the lack of an intermediate level of description. In the field of artificial intelligence (AI), the early attempts to create neural networks had to be abandoned because the early 2-layer networks could not process information usefully (eg, interpret images or translate languages). It took more than 30 years to understand that neural networks needed an intermediate layer to process information usefully and to implement this intermediate layer [22,23]. The Research Domain Criteria initiative of the NIMH has attempted to bridge this gap with limited success to date [24]. Technological advances over the past decade offer a promising approach based on new measures that may be essential for understanding mental disorders and predicting their trajectories. Several new tools allow us to continuously monitor objective behavioral measures (eg, hours of sleep) and densely sample subjective measures (eg, mood). The promise of this approach, referred to as digital phenotyping was recognized almost a decade ago [25], with its potential impact on psychiatry being compared to the impact of the microscope on biological sciences [26].

However, simply gathering a large amount of informative data about a single patient is not helpful by itself. Just as a clinician struggles to synthesize the information from over 100 clinical notes and dozens of laboratory reports available in an electronic health record, the massive amount of data provided by digital phenotyping is useless unless these data can be properly analyzed in a clinical context and with the proper statistical tools. Machine learning, by extracting complex patterns from multiple sources of high-dimensional time-varying data [27], is an ideal tool to address this problem [28-30]. Nonetheless, some challenges with machine learning still need to be addressed before it can be used to make actionable predictions in psychiatry. These challenges include unreliable inherent assumptions [31,32], model instability [33], and lack of interpretability [34] or explainability [35] of results (the black box problem).

Despite the intuitive belief that collecting densely sampled data (big data) improves clinical outcomes, recent clinical trials have not shown that incorporating digital phenotyping improves clinical outcomes [36-40]. This is an example of the so-called “AI chasm,” which refers to the gap between developing algorithms and their actual real-world implementation and clinical impact [41]. As discussed above, some reasons for this chasm include the disconnect between building good individual predictive models for the broader population and making individual inferences [42]. Bayesian procedures offer a potential solution to link inferences and predictions [43]. Other simpler reasons to address include the lack of expertise needed to implement tools into clinical practice [44], poor data quality compromising the reliability and accuracy of models [45-47], and a lack of standardization [38].

During the next decade, achieving clinically actionable predictions in psychiatry will require a stepwise development and implementation approach [12], similar to the successful methods used in other medical fields, such as predicting and preventing cardiovascular disease [48]. The first step will be to identify individual digital measures of objective behaviors and subjective mental states (digital markers) that can be integrated with sociodemographic data, clinical characteristics, and biomarkers to create multimodal signatures that predict clinical outcomes (akin to risk scores in other medical fields). These multimodal signatures will need to be reliably and accurately associated with individual clinical states and trajectories. Validation studies will require cohort studies with adequate sample sizes and sufficient duration to generate enough analyzable clinical events [49]. We foresee that different multimodal signatures will be used for detection (diagnosis) and prediction (prognosis). Once the reliability, specificity, and sensitivity of these multimodal signatures are established, prospective randomized clinical trials (RCTs), with adequate sample sizes, complemented with real-world observation studies, will need to demonstrate that they can be used to tailor the treatment of individual patients and improve outcomes. Albeit costly, these RCTs are needed to fulfill the promise of clinically actionable predictions leading to individualized, timely treatment. We are also mindful of a recent review [50] that emphasized methodological challenges in RCTs investigating smartphone-based treatment interventions for mental disorders, including lack of trial registrations, inappropriate comparators, lack of blinding, selection bias, and lack of generalizability.

In parallel, the incorporation of digital phenotyping, first in RCTs and later in clinical practice, will require addressing complex ethical issues raised by the intense monitoring of behavior and mental state, which some people may consider too invasive regardless of its potential benefits [51]. This work can be informed by lessons learned from other fields [52]. To prepare for the deployment of the new decision-making tools we foresee and to understand their potential and pitfalls [53], we will need to start training in medical schools and continue training throughout our professional life [54].

In conclusion, we believe that technological advances, in the context of a more holistic approach that considers all determinants of health, will allow us to create individual multimodal signatures for early detection and personalized intervention for mental disorders. However, this potential transformation in psychiatry will require another decade of investment and effort to become a reality.

## Abbreviations

AI: artificial intelligence
NIMH: National Institute of Mental Health
RCT: randomized clinical trial
